# Novel Utilization of Protrieve™ to Decrease Embolization Risk During Radical Nephrectomy With Inferior Vena Cava (IVC) Thrombectomy: A Case Report

**DOI:** 10.7759/cureus.68185

**Published:** 2024-08-30

**Authors:** Samantha Cooper, Gabrielle R Yankelevich, Harry St. Clair Clarke, Angelo Lin

**Affiliations:** 1 Department of Urology, Philadelphia College of Osteopathic Medicine, Philadelphia, USA; 2 Department of Urology, Medical University of South Carolina, Charleston, USA; 3 Department of Transplant Surgery, Medical College of Georgia, Augusta University, Augusta, USA

**Keywords:** clear cell renal cell carcinoma, tumor emboli, radical nephrectomy with ivc thrombectomy, ivc thrombectomy, open nephrectomy, ivc thrombus

## Abstract

Intraoperative efforts have been made to reduce the risk of tumor thrombus in renal cell carcinoma during nephrectomy and inferior vena cava (IVC) thrombectomy to decrease mortality. We present a patient diagnosed with renal malignancy and IVC thrombus. The patient underwent a nephrectomy combined with IVC thrombectomy, utilizing a novel approach with the Inari Protrieve™ (Inari Medical, Inc., Irvine, CA) to prevent embolization of tumor thrombus. With the integration of Protrieve™, we propose an approach to facilitate future surgical techniques for nephrectomy with IVC extension.

## Introduction

Renal cell carcinoma (RCC) accounts for nearly 4% of all malignancies [1]. Patients can be diagnosed at any stage, typically with either vague or no symptoms. If the patient exhibits the standard triad of hematuria, flank pain, and abdominal tumor, they are more likely to receive an early diagnosis, which can decrease the likelihood of developing venous extension [2]. However, RCC often lacks these early warning signs, leading to nearly 30% of cases diagnosed at advanced or metastatic stages [3]. Stage T3 RCC with venous extension can form a tumor thrombus, with approximately 10% of these cases leading to invasion of the inferior vena cava (IVC) [4].

Intraoperative efforts have been made to reduce the risk of tumor thrombus in RCC during nephrectomy and IVC thrombectomy. It has been noted that complete removal of the extension of the tumor thrombus into the IVC does not affect that patient's prognosis. However, failure to remove has been associated with poor survival rates [5,6]. Regardless of preoperative or intraoperative efforts, the standard surgical procedures for the removal of RCC and tumor thrombus are associated with increased mortality rates of 2%-13% [7].

We present a patient with RCC along with an IVC thrombus. The patient underwent nephrectomy with IVC thrombectomy utilizing a novel approach with the Inari Protrieve™ (Inari Medical, Inc., Irvine, CA) to prevent embolization of the tumor thrombus.

## Case presentation

A 65-year-old male with a medical history of prior tobacco use, hypertension, hyperlipidemia, obesity (BMI of 37), and non-insulin-dependent diabetes mellitus presented to the urology clinic after he received imaging for the left arm and abdominal pain, which showed evidence of metastatic disease. Imaging revealed a 14-cm left renal mass with level III thrombus, left adrenal metastasis, left humeral lesion, and T12 lytic lesion. He underwent a renal biopsy, which showed grade 2 clear cell renal carcinoma with granular features. He was presented at the multidisciplinary tumor board and was recommended to undergo systemic therapy for presumed metastatic RCC.

He underwent humeral lesion resection and received orthopedic stabilization to mitigate the risk of an impending pathologic fracture. The pathology results revealed a positive diagnosis of plasma cell neoplasm, which led to a positive multiple myeloma workup. Medical oncology and radiation oncology discussed that the vertebral lesion was most likely multiple myeloma and recommended radiation to this lesion. Given this, he did not undergo systemic chemotherapy for RCC and returned to urology for treatment after radiation was completed.

**Figure 1:**
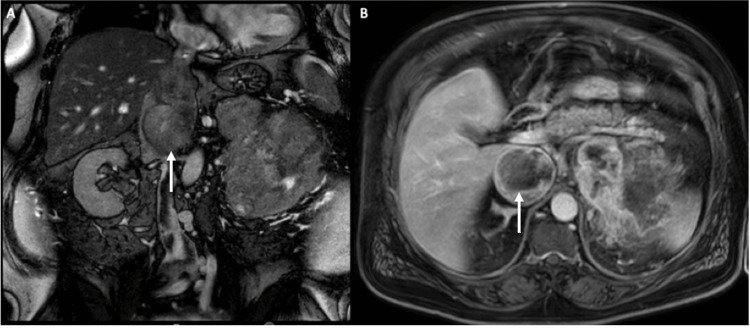
MRI showing coronal (A) and axial (B) views of the left renal mass with thrombus (white arrows) invading the left renal vein and up to hepatic confluence in the IVC MRI: magnetic resonance imaging; IVC: inferior vena cava

The repeat imaging was obtained, and it showed no changes from the previous images. The patient's case was deliberated at the multidisciplinary tumor board, and it was recommended that he undergo systemic therapy, considering his intricate oncologic history, including two primary malignancies. The genitourinary medical oncologist, malignant hematologist, and the patient's local primary oncologist approved his systemic therapy plan. The plan consisted of neoadjuvant lenvatinib and pembrolizumab for RCC, as well as daratumumab for multiple myeloma. He was planned for four cycles of pembrolizumab and then reimaging to assess response for candidacy for nephrectomy with IVC thrombectomy. His imaging showed stable renal and IVC pathology with improvement of multiple myeloma lesions (Figure 1). He consulted with the urology department, and after receiving preoperative clearance from anesthesia, hematology, and oncology, he was scheduled for an open left nephrectomy with IVC thrombectomy. A left renal artery embolization was planned for the day prior, given multiple parasitic vessels. His preoperative creatinine was 1.6 mg/dL, with an estimated glomerular filtration rate (eGFR) of 48 mL/min/1.73 m^2^.

The patient underwent an uncomplicated left renal artery embolization and was admitted overnight with an epidural for pain control. The following morning, he underwent a left open nephrectomy, resulting in an estimated blood loss of approximately 2,100cc. The surgery took approximately seven hours, and he received 10 L of crystalloid and three units of blood.

Briefly, the urology team started with a midline incision and mobilized the kidney. After the complete mobilization of the kidney from the retroperitoneum, the transplant surgery team assisted in isolating and ligating the left renal vein and renal artery to complete the nephrectomy. The left renal vein was divided to the right of the aorta and oversewn. The infrarenal IVC, right renal vein, and infrahepatic IVC were encircled with vessel loops in a Potts tie fashion for vascular control. The portal hepatic vein was encircled with umbilical tape and passed through a Rummel tourniquet for the Pringle maneuver. Finally, we placed a vessel loop around the suprahepatic IVC at the diaphragm level.

A Protrieve™ device was placed in the right internal jugular vein, and the device was deployed right at the superior vena cava diaphragmatic junction under fluoroscopic guidance. Protrieve™ is a device designed by Inari to prevent pulmonary embolism during interventional thrombectomy of the iliac or IVC. We previously had a case of pulmonary embolism for a similar situation and decided that this would be a beneficial addition to prevent potential complications.

**Figure 2:**
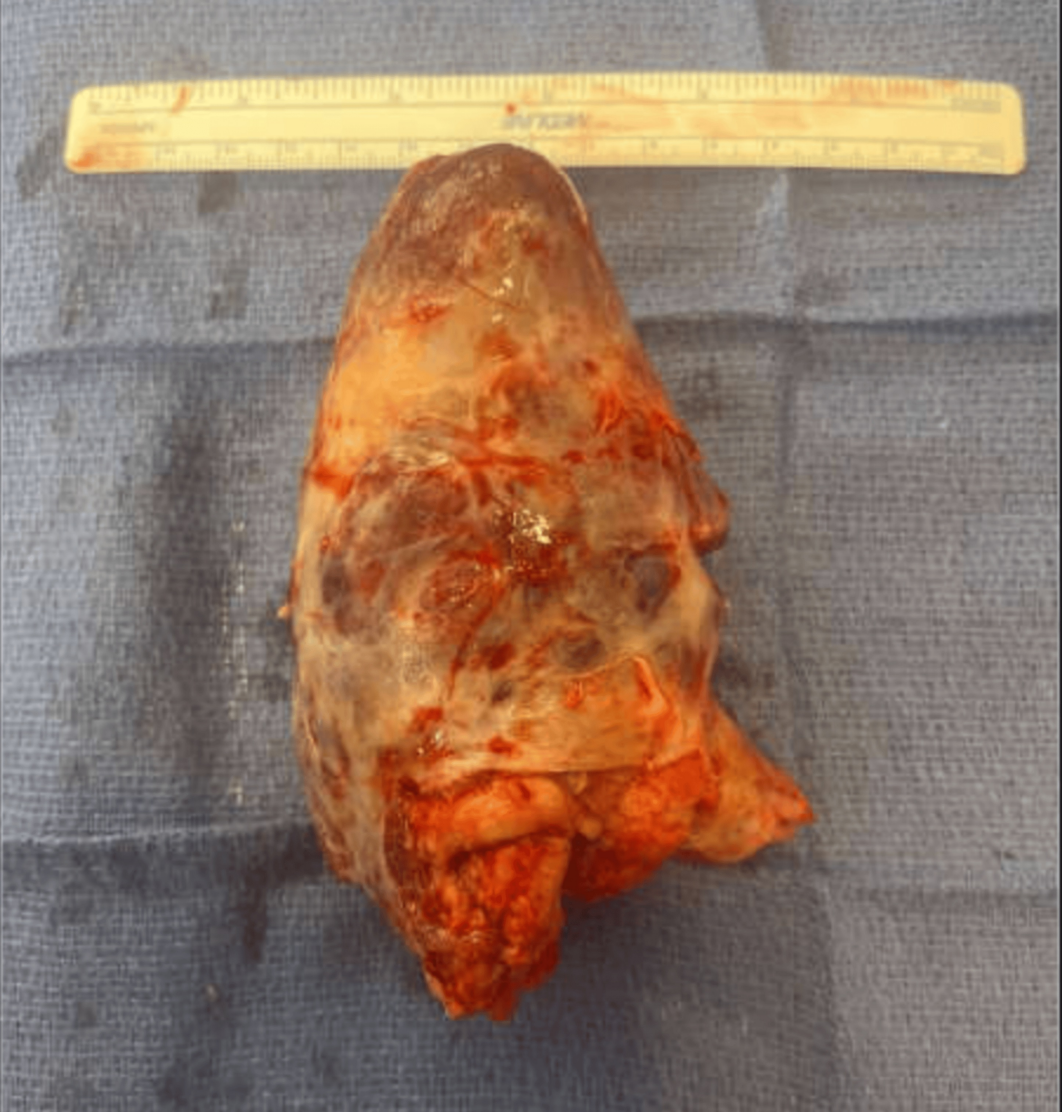
Large burden of intact intracaval thrombus removed

Once vascular control was obtained by tightening the vessel loops or clamping, a venotomy was made extending from the left renal vein stump to the infrahepatic IVC. Using a vein elevator and blunt finger dissection, the tumor inside the IVC was removed in one piece (Figure 2). The infrahepatic IVC was occluded, so we released the suprahepatic and portal clamps to restore the portal flow. The IVC venotomy was closed with 5-0 Prolene. The flow was reestablished in the IVC by releasing the rest of the vessel loops. The Protrieve™ was removed without any issues. The neck site was closed with a figure-of-eight pattern using 3-0 nylon sutures, which is our standard procedure for achieving hemostasis at our institution. This procedure has a bleeding risk similar to that of a central line.

The surgical procedure was ultimately successful, and we demonstrated that Protrieve™ was an effective adjunct in preventing tumor emboli. With the addition of Protrieve™, we propose the following approach to facilitate future surgical techniques for nephrectomy with IVC extension. The detailed steps are listed in Table 1.

**Table 1:** Novel process for left nephrectomy with IVC thrombus up to hepatic vein confluence IVC: inferior vena cava; IJ: internal jugular

Step number	Description
1	Prep and drape with split sheets to include the right neck and groin in the field. Use a large Bookwalter retractor for exposure
2	Start with Kocher maneuver to expose the IVC and aorta
3	Identify and encircle the left renal vein above aorta and, if possible, identify the renal artery for ligation
4	Divide and over-sew the renal vein with 3-0 Prolene and ligate the renal artery if unsuccessful on the previous step
5	Identify and encircle the right renal vein with umbilical tape and red rubber
6	Mobilize the liver and divide the short hepatic veins to get adequate cava length above the renal vein for clamping if needed
7	Encircle the IVC with umbilical tape and a red rubber at the diaphragm below the diaphragmatic veins. If possible, encircle the IVC below the hepatic veins
8	Encircle the liver hilum with umbilical tape and a red rubber for the Pringle maneuver
9	Access the right IJ (consider having anesthesia place a right IJ catheter at the beginning of the case) and place the Protrieve™ under fluoroscopy and ultrasound guidance on the IVC at the diaphragm. Make sure the tip is just above the diaphragm when deploying the basket. Put an IV Tegaderm under the sheath first before putting the large Tegaderm on top to secure the sheath. Consider shooting a fluorogram to confirm Protrieve™ placement
10	Sequentially tighten the infra-IVC, hepatic hilum (if supra-IVC umbilical tape is above hepatic vein), and then supra-IVC
11	Cut open the renal vein and extend transversely across the IVC. Mobilize bluntly using finger dissection circumferentially
12	If unable to reach the tip of the thrombus superiorly, then make a transverse incision on the cava below the hepatic veins and use blunt finger dissection to free up the tip of the thrombus. Remove the thrombus. Flush the cava with a red rubber catheter, then get temporary control of the cava venotomy with two 3-0 Prolene U-stitches
13	Clamp the infra-hepatic cava above venotomy. Release supra-IVC and then hepatic hilum to return portal circulation
14	Utilize ultrasound and/or venogram to ensure removal of thrombus in the retrohepatic vena cava if the thrombus did not come out in one piece
15	Repeat 10-14 if needed to remove all retrohepatic thrombus. Consider getting control of IVC below the hepatic vein to avoid repeated Pringle maneuver
16	Mobilize the right lobe of liver by dividing the short hepatic veins to allow access to the retrohepatic IVC if necessary in order to remove the residual thrombus
17	Close the inferior cava venotomy
18	Release the inferior IVC and right renal vein
19	Ultrasound the IVC to ensure no residual thrombus
20	Aspirate the Protrieve™ to remove any thrombus in the basket before retrieving the basket and removing the sheath. Alternatively, one can try using the FlowTriever® suction device (Inari Medical, Inc.) to aspirate the residual thrombus under fluoroscopy and/or ultrasound guidance through the Protrieve™ device. Place a 2-0 or 3-0 nylon on the skin for hemostasis and hold pressure for 5-10 minutes
21	Proceed with left nephrectomy. This will make the nephrectomy much easier in a medial to lateral fashion since the vascular control was obtained already in steps 3 and 4

## Discussion

Results

Postoperative CT imaging showed a bland thrombus extending from the proximal bilateral common iliac veins to the IVC up to the level of the liver for which the patient underwent IVC filter placement with interventional radiology. The final pathology was pT4N0 with grade 3 clear cell carcinoma directly invading the adrenal gland and extending into the renal sinus, 11 benign lymph nodes, and tumor thrombus with necrosis. He was presented at the multidisciplinary board and was recommended to have adjuvant pembrolizumab. He was last seen five months after the operation. His creatinine was 1.6 mg/dL, with an eGFR of 48 mL/min/1.73 m^2^, which was stable from his preoperative creatinine. He underwent an IVC venogram with interventional radiology, noting a stable long-segment chronic occlusion of the infrarenal to suprarenal IVC with collateralization. There was no evidence of acute or subacute thrombus to warrant mechanical thrombectomy, and they performed a successful IVC filter removal.

Comment

We present the use of a sheath to prevent embolization of tumor thrombus during IVC thrombectomy. Intraoperative tumor embolization during a nephrectomy IVC thrombectomy is reported to occur between 1.8% and 5.3%, with a high mortality of 25%-60% if this occurs [8]. The benefits of this approach are that only 34.9% of retrievable IVC filters placed for a temporary indication are removed according to a systematic revieẉ. However, in our case, this is removed intraoperatively since it is placed by the surgeon [9]. The Protrieve™ device allows surgeons to prevent pulmonary embolism and also enables the removal of residual clots percutaneously without significant blood loss.

## Conclusions

We propose using a novel approach for nephrectomy and IVC thrombectomy with the Inari Protrieve™ to prevent embolization of tumor thrombus. Ultimately, it is important to find surgical techniques that can reduce the incidence of thrombus and improve mortality in future patients, which we have found success by using our novel method.
